# Metastasis in neuroblastoma: the *MYCN* question

**DOI:** 10.3389/fonc.2023.1196861

**Published:** 2023-05-18

**Authors:** Swapnil Parashram Bhavsar

**Affiliations:** Pediatric Research Group, Department of Clinical Medicine, Faculty of Health Sciences, UiT - The Arctic University of Norway, Tromsø, Norway

**Keywords:** MYCN, oncogene, poor prognosis, metastasis, neuroblastoma

## Abstract

Oncogenic drivers like *MYCN* in neuroblastoma subsets continues to present a significant challenge owing to its strong correlation with high-risk metastatic disease and poor prognosis. However, only a limited number of MYCN-regulatory proteins associated with tumor initiation and progression have been elucidated. In this minireview, I summarize the recent progress in understanding the functional role of *MYCN* and its regulatory partners in neuroblastoma metastasis.

## Introduction

## Metastasis

Genetic changes are acquired by rare cells in the primary tumor mass which confer them dominant phenotypes like the ability to resist growth inhibiting signals, avoiding apoptosis, acquisition of constitutive mitogenic signals and induction of angiogenesis. A subset of these rare cells harbors yet more mutations that increases the propensity to metastasize to distant organs and seed new colonies (1). This process termed - tumor metastasis, is the main cause of cancer related deaths and is the most important obstacle in the treatment of cancer patients. Despite decades of research and improvements in treatment approaches thereof, the survival rates of patients with metastatic disease are very low. For example, the overall survival of high-risk neuroblastoma patients remains around 50 - 60% (2). Therefore, there is an immediate need to better understand the molecular mechanisms underlying tumor metastasis to develop novel and effective therapeutics to improve the quality of cure in this vulnerable population.

## Neuroblastoma

The most common pediatric cancer of the developing sympathetic nervous system – Neuroblastoma, as coined by James Wright in 1910 (3), after a century of research is still enigmatic due to its extremely heterogenous clinical nature. It has been demonstrated to range from an aggressive metastatic disease with poor prognosis to spontaneous regression or differentiation into benign histological variants (4). This explains the highly variable clinical behavior of neuroblastoma tumors, some of which are easily treatable while majority of them are aggressive (5).

Despite technological advances, the understanding of the genomic features of neuroblastoma is still modest. Numerical and segmental chromosomal abnormalities, transcriptomics, amplifications and/or overexpression of specific genes, variations in ploidy and epigenetics have all proven to play a critical role in neuroblastoma pathogenesis (6, 7). The most frequently harbored alterations of specific genes in neuroblastoma include heritable mutations in the *ALK* (6) and *PHOX2B* (8) observed in familial neuroblastoma, chromatin remodeling genes like *ATRX* (6), *ARID1A* and *ARID1B* (9) and other important genes like *PTPN11*, *MYCN*, and *NRAS* (6). In addition, cytogenetic abnormalities like gain of the long arm of chromosome 17 (17q gain) (10), 11q loss of heterozygosity (LOH) and 1p deletion (11) are also observed in neuroblastoma. Interestingly, all these aberrations are predictor of poor outcome in neuroblastoma (12).

The *MYCN* gene in neuroblastoma subsets continues to present a significant challenge. It is deregulated in both the pediatric and adult cancers (13). Multiple studies have demonstrated *MYCN* to function as an oncogenic driver in neuroblastoma. This is mainly proven by the guided ectopic expression of *MYCN* (alone or in combination of *LMO1* or *ALK*) in specific cell lineages of zebrafish models (14, 15) or genetically engineered mouse models (GEMM) (16, 17) which results in the development of neuroblastoma. *MYCN* as a transcription factor, activate and/or repress various genes involved in cell proliferation, survival, and apoptosis ultimately leading to increased cellular self-renewal capacity, apoptotic resistance, and metabolic flexibility (18). Amplification of *MYCN* indicates aggressive or high-risk metastatic disease and poor patient prognosis (19). Nearly half of all the neuroblastoma patients at diagnosis show metastasis (20). The most frequent sites of metastasis are in bone marrow (70.5%), bone (55.7%), lymph nodes (30.9%), liver (29.6%), and intracranial and orbital sites (18.2%) (21). Abnormal *MYCN* expression is present at diagnosis and is never acquired during later tumorigenesis of MYCN-non-amplified neuroblastoma (18) which might be the reason behind observation of metastasis in neuroblastoma patients at diagnosis.

## MYCN

Discovered in 1983, *MYCN* (22, 23), belongs to a Myc family of genes which consists of c-myc (MYC), l-myc (MYCL) and n-myc (MYCN) (24). It is located on chromosome 2p24.3 (25) and its normal function include regulation of the cell cycle and apoptosis (26). The *MYCN* gene in turn is regulated by different mechanisms including and not limited to epigenetic regulation, miRNAs, and formation of G-quadraplexes (27). It is commonly amplified and overexpressed in a cancer setting (28). And it contributes to multiple facets of metastasis like adhesion, motility, invasion, and degradation of surrounding matrices (19). Molecular processes central to the *MYCN’s* oncogenic activity include the hallmarks of cancer influencing the cell cycle, apoptosis or cell death and cellular metabolism reviewed elsewhere (29). It is thus, an important genetic marker for prognosis, diagnosis, and therapeutics.

*MYCN* is extensively studied in neuroblastoma. It is amplified in 25% of all neuroblastomas and nearly 50% in high-risk neuroblastoma cases (30). It is the best characterized negative prognostic indicator that not only stratifies risk in neuroblastoma but also predict poor clinical outcome. Therefore, overexpression of *MYCN* is associated with poor prognosis, advanced stage of disease, rapid tumor growth and metastasis (31). *MYCN* thus plays a significant role in neuroblastoma biology and is the most important target for therapy.

## The *MYCN* regulation in metastatic neuroblastoma

**Figure 1:**
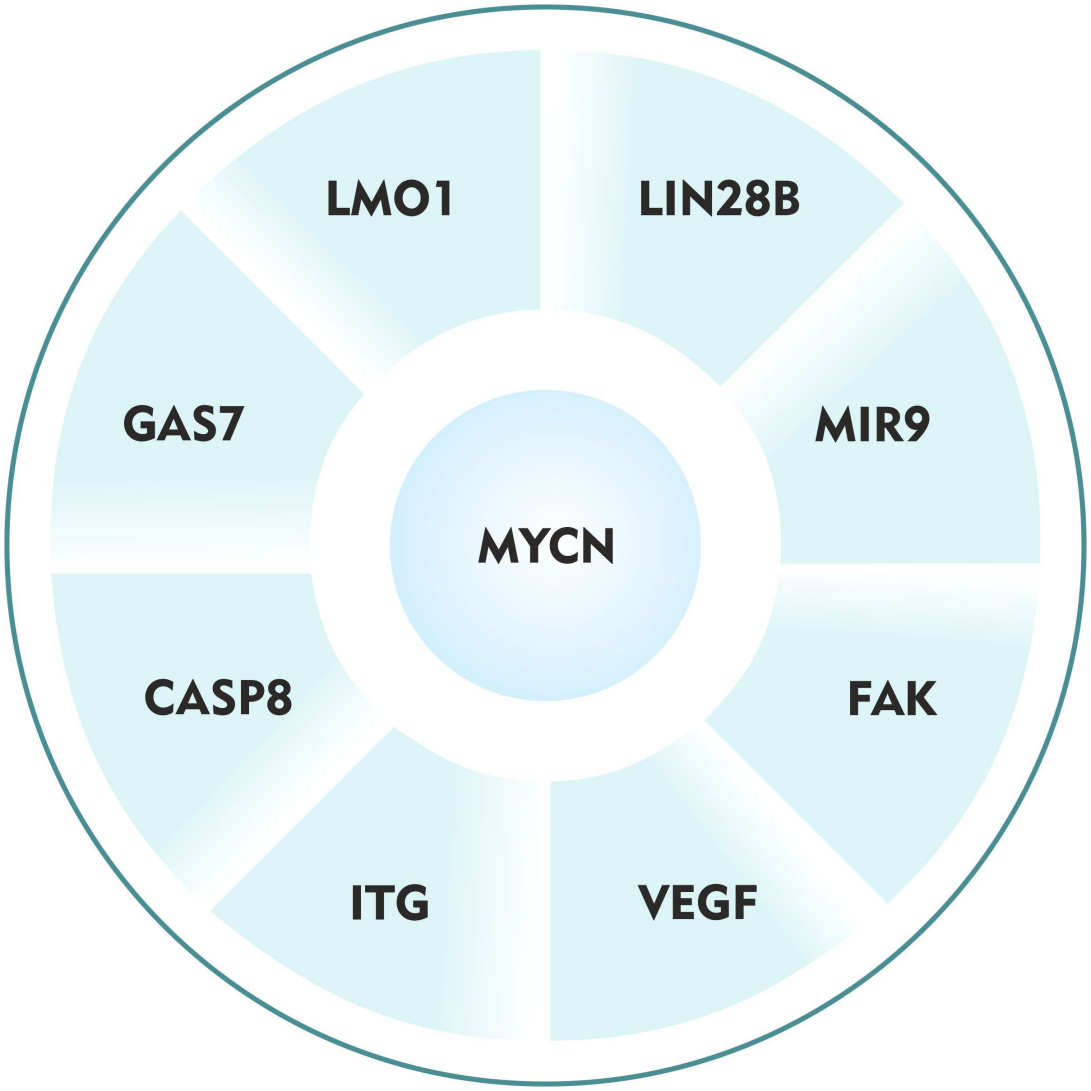
The ‘Metastatic wheel of neuroblastoma’ driven by MYCN oncogene.

The interplay of the host genetic factors, somatic mutations, chromosomal abnormalities, and epigenetic alterations lead to highly aggressive metastatic neuroblastoma (6). But the fundamental molecular mechanisms remain to be elucidated. Very few studies have demonstrated a crucial role of *MYCN and* its regulatory proteins in neuroblastoma metastasis (**Table 1**). Investigating these thoroughly, I define here, the ‘*Metastatic wheel of neuroblastoma*’ driven by *MYCN* oncogene (**Figure 1**). I explored eight different *MYCN* regulatory networks and sought to determine the mechanisms by which this oncogene influences neuroblastoma metastasis.

**Table 1:** The articles investigating the role of *MYCN* and its regulatory genes in neuroblastoma.

Model system	Regulatory partners of *MYCN*	Functional effects in neuroblastoma	References
Cell lines, Zebrafish & Mice	*GAS7*	Invasion, Migration & Metastasis	32
Cell lines & Mice	*LIN28B*	Invasion, Migration & Metastasis	33
Cell lines & Zebrafish	*LMO1*	Invasion, Migration & Metastasis	15
Cell lines & Mice	*MIR9*	Invasion, Metastasis & Angiogenesis	34 & 35
Cell lines & Mice	*FAK*	Invasion, Migration & Metastasis	36 & 37
Cell lines	*ITG*	Invasion & Migration	38
Cell lines & Mice	*VEGF*	Angiogenesis	39
Cell lines & Mice	*CASP8*	Metastasis	40 & 41

## Growth arrest specific 7

*GAS7* is an evolutionary conserved gene which play key role in cell structure and motility. It has been associated with multiple development pathways and recently characterized as a metastasis suppressor gene (42). Dong and colleagues have reported the first genetic evidence linking *GAS7* deficiency and metastasis in MYCN-driven neuroblastoma. Chromosome 17p13.1 harbors *GAS7* and *TP53* genes. And they observed a heterozygous deletion of 17p arm in high-risk cases. Bioinformatics analyses revealed association of 17p deletion with poor clinical outcome. Moreover, they also observed significantly lower level of *GAS7* expression in MYCN-amplified vs. MYCN-non-amplified cases. This prompted them to consider if *MYCN* binds to *GAS7* promoter directly. But they did not observe any such binding. However, by dual cross-linking ChIP-PCR assay they showed that *MYCN* interacts with SP1 transcription factor, by forming a transcription repression complex at the *GAS7* promoter region to regulate *GAS7* activity indirectly. Next, using both zebrafish and mammalian model systems, they show that reduced expression of the *GAS7* promotes metastasis of MYCN-amplified neuroblastoma cells (32). Thus, loss of *GAS7* is one of the main events driving metastasis in neuroblastoma.

## Lin-28 homolog B

LIN28 and LIN28B are highly conserved RNA-binding proteins associated with advanced human malignancies (43). Activation of these proteins is correlated with poor clinical prognosis (44). Molenaar et al., has demonstrated an oncogenic role of *LIN28B* in neuroblastoma. They reported that *LIN28B* is genomically altered and overexpressed in high-risk neuroblastoma and it’s correlated with adverse clinical outcome (45). Interestingly, the role of *LIN28* and *LIN28B* in blocking the maturation of the tumor suppressor microRNA *let-7* family is well established (46). Since, the 3’ UTRs of *MYC* and *MYCN* harbor *let-7* binding sites, repression of *let-7* results in higher expression of *MYC* and *MYCN*. Thus, the genetic loss of *LIN28B* in *MYCN*-driven neuroblastoma results in loss of metastatic potential both *in vitro* using MYCN-amplified neuroblastoma cell lines and/or PDX samples and *in vivo* using immunocompromised mice (33). Therefore, the role of *LIN28B* in neuroblastoma initiation and its correlation with *MYCN* expression makes it an important therapeutic target for intervention. Taken together, these studies highlight the significant role of *LIN28B* and *MYCN* in metastasis.

## LIM domain only 1

*LMO1* gene is a member of LMO protein family, which includes *LMO1*, *LMO2*, *LMO3* and *LMO4*. These proteins have been shown to play an oncogenic role in types of cancer including neuroblastoma (47). Disease-associated single nucleotide polymorphisms (SNPs) are often located within the super-enhancer elements (48). Oldridge and colleagues identified SNP within a super-enhancer element in the intronic region of *LMO1* which led to the high expression of *LMO1* and neuroblastoma pathogenesis (49). Further to this study, Zhu et al., using a novel zebrafish model, demonstrate how *LMO1* and *MYCN* genes cooperate to first initiate neuroblastoma and further contribute to metastatic disease progression. They generated a stable transgenic zebrafish model that overexpress *LMO1* gene in the peripheral sympathetic nervous system (PSNS). They observed that overexpressing *LMO1* alone do not develop neuroblastoma but its overexpression along with increased *MYCN* expression led to enhanced neuroblastoma initiation and disease penetrance. Interestingly, they also observed distant metastasis in the transgenic fish overexpressing both *MYCN* and *LMO1*. This observation was supported by the increase in expression of panel of genes involved in tumor cell-extracellular matrix interactions like *LOXL3* and integrins - *ITGA2B*, *ITGA3*, and *ITGA5* (15, 50). Thus, these findings confirm the important role of *MYCN* and *LMO1* in promoting neuroblastoma initiation, progression, and metastasis.

## MicroRNA-9

The ubiquitous involvement of microRNAs in shaping the cellular properties have paved way to speculate its role in influencing metastasis. Thus, multiple microRNAs have demonstrated its role either in promoting or repressing metastasis in different cancers. However, the relation between miRNA and *MYCN* in inducing metastasis is few explored in neuroblastoma. Interestingly, in breast cancer, studies by Ma et al., showed that *miR-9*, a microRNA induced by *MYC/MYCN*, targets E-cadherin, and not only primes cancer cells for epithelial to mesenchymal transition (EMT) and invasion, but also contribute to promoting angiogenesis. Thus, leading to growth, proliferation, invasion, and migration of cancer cells and hence metastasis. However, this *miR-9* effect is not observed in neuroblastoma since the tumors do not express E-cadherin (34, 51). Nonetheless, studies by Zhang et al., showed that *miR-9* expression was downregulated in neuroblastoma cell lines and primary tissues. They also observed an inverse correlation between *MMP14*, a matrix metalloproteinase playing a critical role in tumor metastasis and angiogenesis, and endogenous *miR-9* expression. Further bioinformatic and functional assays confirmed *MMP14* as a target of *miR-9*. The effect of targeting *MMP14* resulted in suppression of invasion, metastasis, and angiogenesis of neuroblastoma cells both *in vitro* and *in vivo* (35). This study was performed using MYCN-non-amplified neuroblastoma cell lines SH-SY5Y and SK-N-SH. Therefore, the role of *MYCN* in inducing *miR-9* expression was not addressed.

## Focal adhesion kinase

FAK is a cytoplasmic protein tyrosine kinase, a crucial signaling component activated by numerous stimuli, which play a key role in normal and tumor cell migration (52). *FAK* is shown to be overexpressed and activated in several advanced-stage solid cancers and promotes tumor progression and metastasis (53). Beierle and colleagues observed upregulation of *FAK* in MYCN-amplified and overexpressed neuroblastoma cell lines and in the advanced-stage human neuroblastoma tumor specimens. This observation prompted them to investigate if *MYCN* regulates FAK expression. Interestingly, bioinformatic analysis revealed two *MYCN* binding sites in the *FAK* promoter region. Moreover, series of *in vitro* and *in vivo* assays confirmed the binding of *MYCN* to sites in the *FAK* promoter. Conditional expression of *MYCN* or inhibition of *FAK* expression resulted in decreased cell viability and increase in apoptosis. Thus, this study clearly demonstrated *MYCN* regulation of *FAK* expression (36, 54). Furthermore, similar group set out to determine if the abrogation of *FAK* would result in alteration of metastatic potential of neuroblastoma cells. As expected, RNA interference-mediated silencing and/or small molecule inhibitors inhibition of *FAK*, decreased the metastatic properties like invasion and migration *in vitro* and decrease the ability of neuroblastoma cells to form metastasis in a nude mouse model *in vivo* (37).

## Integrins

Integrin signaling is crucial in regulating multiple functions in normal and transformed cells. Increased expression of integrins (e.g., αvβ3, αvβ5, α5β1, α6β4, α4β1 and αvβ6) is correlated with proliferation survival, invasion, and metastasis in various cancers (55). Study by Wu and colleagues showed that α4β1 and α5β1 integrins were expressed in the late stage of neuroblastoma tumors and cell lines. To find out how integrins initiated neuroblastoma motility, they performed knockdown and reconstitution experiments. Whereas knockdown of α5β1 led to FAK/Src/p130Cas dependent neuroblastoma motility, knockdown of α4β1 led to Src/p130Cas dependent neuroblastoma motility but not *FAK* (56). In another study, Tanaka et al., observed that MYCN-non-amplified cell lines (SK-N-SH and NB69) had higher expression of integrin α1 whereas MYCN-amplified cell lines (IMR-32, NB1, NB9, and NB19) had its lower expression. Therefore, knockdown of *MYCN* in NB1 and NB19 led to increased expression of integrin α1 and reduced migration. And overexpression of *MYCN in* SK-N-SH and NB69 led to decreased expression of integrin α1 and enhanced migration (38). This observation thus suggests that *MYCN* promotes neuroblastoma metastasis by downregulating integrin α1.

## Vascular endothelial growth factor

*VEGF* is a key regulator of the physiological and pathological angiogenesis, a highly complex and coordinated process for the formation of the new blood vessel growth and maturation (57). *VEGF* inhibition is a strategy for the prevention of angiogenesis and its inhibitors are undergoing clinical trial in several malignancies (58, 59). Kang et al., showed that tumor angiogenesis correlates with increased expression of the *VEGF* and poor clinical outcome in neuroblastoma. And they proposed that *MYCN* plays a significant role as a novel effector of PI3K-mediated regulation of *VEGF* and hence tumor angiogenesis, in a highly vascularized, malignant neuroblastoma (39). The observation that *PI3K* inhibition reduce tumor growth of murine neuroblastoma model (60) prompted Kang and colleagues to check its effect on angiogenic capacity of human neuroblastoma in mice bearing neuroblastoma xenografts. Selective inhibition of *PI3K* in this regard led to reduction of *VEGF* expression and secretion and reduced growth of established neuroblastoma tumors. Next, they investigated if *MYCN* inhibition could also regulate the *VEGF* expression in neuroblastoma cells. Interestingly, siRNA knockdown of *MYCN* in MYCN-amplified neuroblastoma cells led to significant decrease in *VEGF* expression and no difference in MYCN-nonamplified cells. This observation indicated the role of *MYCN* in the regulation of *VEGF* expression (39). Taken together, these findings indicate the important role of *MYCN* in the underlying molecular mechanisms of PI3K/MYCN/VEGF regulation of critical angiogenic pathways in neuroblastoma.

## Caspase-8

In 2000, Teitz et al., in a seminal paper proposed that Caspase-8 is deleted or preferentially silenced through epigenetic mechanisms in MYCN-amplified neuroblastomas. This observation indicated that Caspase-8 act as a tumor suppressor in neuroblastoma (40, 61). *CASP8* gene is mapped to chromosome 2q33. Takita and colleagues demonstrated a common region of allelic imbalance on chromosome 2q and an alteration of *CASP8* in neuroblastoma. They concluded that epigenetic silencing and allelic imbalance are two important mechanisms for the inactivation of Caspase-8 in neuroblastoma (62). Further studies in this regard led Cui and colleagues to establish a link between *MYCN* and the death receptor apoptotic pathways (involves Caspase-8) in neuroblastoma (63).

In 2006, Stupack and colleagues, were successfully able to demonstrate for the first time the role of Caspase-8 in metastases. They provided evidence that Caspase-8 act as a as a metastasis suppressor gene and regulated the survival and invasive capacity of neuroblastoma cells (64). This study was further complimented with yet another publication from Teitz et al., establishing the role of Caspase-8 in metastasis. In this study, they established an immunocompetent mouse model for metastatic neuroblastoma which recapitulated not only overexpression of *MYCN* but also loss of Caspase-8 expression. Microarray expression studies from the mouse primary tumors revealed genes involved in epithelial to mesenchymal transition (EMT) and Extracellular Matrix (ECM) (41). Thus, given the necessity of animal models in testing therapies for metastatic neuroblastoma, Th-MYCN/caspase-8–deleted mouse could serve as an important model system.

## Treatment

Due to the clinical and biological heterogeneity of neuroblastoma tumors, the treatment approaches tailored are mainly based on low, intermediate, and high-risk stage groups. Children with low and intermediate-risk get surgical tumor resection with or without chemotherapy. On the other hand, high-risk patients require intensive multimodal treatment regimens, which is complex and contains multiple consecutive phases (65). However, despite intensive modalities, 50-60% patients with high-risk disease will ultimately relapse with no curative treatment available for these patients (5). Therefore, new alternate approaches are being tested to combat this deadly disease. Recent novel immunotherapy approaches to target neuroblastoma have demonstrated that anti-GD2 monoclonal antibody therapy and CAR T cell therapy for patients with high-risk neuroblastoma improves the event-free and overall survival (66, 67). Given the significant role of *MYCN* in neuroblastoma biology and poor clinical outcomes, novel treatments targeting *MYCN* should be developed for patients with neuroblastoma.

## Discussion

The focus of this review is to explore the novel mechanisms underlying tumor metastasis in MYCN-driven neuroblastoma. The fact that around 50% of the neuroblastoma patients show metastasis with *MYCN* amplifications at diagnosis indicate that this oncogene might have a significant role in promoting metastasis. Decades of research in this regard has led to discoveries of only a couple of regulatory genes of *MYCN* (*GAS7*, *LIN28B*, *LMO1*, *MIR9*, *FAK*, *ITG*, *VEGF* and *CASP8*) having direct or indirect role in facets of metastasis as illustrated in this review. Though some of these studies imply the influence of *MYCN* and its regulatory proteins in metastatic neuroblastoma, very few studies successfully demonstrate its role both *in vitro* and *in vivo* (33, 35, 36). Therefore, advanced bioinformatic techniques and/or high throughput technologies are necessitated to find new candidate genes and its association with *MYCN* in promoting metastasis. Studies in types of cancers have implicated effectors of the EMT (68, 69), which are poorly investigated in neuroblastoma. Thus, either independent or co-operative role of such genes with *MYCN* requires further investigation both *in vitro* and *in vivo*.

Modeling of the pediatric cancer in diverse animal models is necessary to understand the role of *MYCN* in metastasis. Models like the widely used TH-MYCN transgenic mouse model and the orthotopic xenografts of primary human neuroblastomas or cell lines may be of valuable resource for evaluating therapeutics (15, 61, 64, 70–72).

Taken together, considering the metastatic role of *MYCN*, it is an ideal and the most wanted target for cancer therapy. Though *MYCN* is thought ‘undruggable’, due to multiple reasons like its MYC similarity, few known MYCN-interacting proteins, lack of *in vivo* testing of MYCN-targets, lack of structural information on MYCN-protein complexes, and the challenges of using traditional small -molecule inhibitors of protein-DNA and/or protein-protein interactions’ (73), novel alternate approaches to target *MYCN* are warranted to combat this deadly pediatric malignancy. I believe that insights provided in this minireview could help develop new ideas and strategies to counter tumor metastasis in MYCN-driven high-risk neuroblastoma.

## Author contributions

The author confirms being the sole contributor of this work and has approved it for publication.
